# Molecular detection of multiple arboviruses in the city of Goiânia-Goiás-Brazil

**DOI:** 10.1590/0037-8682-0539-2023

**Published:** 2024-09-06

**Authors:** Jordana Farias Corrêa, Silvia Maria Salem-Izacc, Elisângela Gomes da Silva, Adriano Roberto Vieira de Sousa, Gabrielly Regis Abrantes, Marina Machado Santos, Juliana Pires Ribeiro, Marco Tulio A Garcia-Zapata, Natália Santana do Nascimento, Carlos Eduardo Anunciação, Sandra Maria Brunini, Elisângela de Paula Silveira-Lacerda

**Affiliations:** 1 Universidade Federal de Goiás, Instituto de Ciências Biológicas, Goiânia, GO, Brasil.; 2 Universidade Federal de Goiás, Instituto de Patologia Tropical, Goiânia, GO, Brasil.; 3 Universidade Federal de Goiás, Faculdade de Enfermagem, Goiânia, GO, Brasil.

**Keywords:** Molecular diagnosis, PCR, qPCR, Arboviruses, Co-infection

## Abstract

**Background:**

Healthcare systems are currently ill-equipped to diagnose arboviruses rapidly and efficiently or to differentiate between various viruses.

**Methods:**

Utilizing molecular techniques, this study examined arbovirus infections in 459 patients from a public health unit in Goiânia-Goiás, Brazil, a region where arbovirus infection poses a significant public health challenge.

**Results:**

Nearly 60% of the analyzed samples tested positive for at least one arbovirus, and over 10% of the patients were co-infected with more than one virus.

**Conclusions:**

Fast and accurate diagnostic tools are essential for informing public health policy and enhancing epidemiological surveillance.

Arthropod-borne viruses (arboviruses) are a group of RNA viruses that infect and replicate in arthropods, such as *Aedes aegypti*, and can be transmitted to vertebrate hosts, including humans. Arboviral infections are a major public health issue in several countries, particularly in tropical and subtropical regions^1^. Most arboviruses belong to the genera *Alphavirus* (family Togaviridae) or *Flavivirus* (family Flaviviridae). Although these viruses are usually geographically restricted, they can spread to endemic areas and become emerging viruses^2^. Human interference in the environment, ecosystem changes, disorderly urban population growth, globalization, expanding international exchange, and climate change are factors that have contributed considerably to the significant increase in arbovirus infections, such as Dengue virus (DENV), Zika virus (ZIKV), Chikungunya virus (CHIKV)^1^ and Mayaro virus (MAYV)^3^.

Currently, arboviruses account for 30% of infectious disease cases globally, posing the most significant challenge in the humid tropical and equatorial areas of Brazil^4^. To date, no effective antiviral drugs are available to treat diseases caused by arboviruses, except for yellow fever and dengue, for which vaccines exist. Moreover, the simultaneous presence of arboviruses such as DENV, ZIKV, and CHIKV has been documented in various regions worldwide, indicating that co-infection among these viruses is not uncommon. However, the impact of each virus on disease severity and mortality in co-infected patients remains poorly understood^1^.

Viremic and co-infected patients may expose the *Aedes aegypti* mosquito to multiple viruses simultaneously. Consequently, the ability of this vector to co-infect and transmit arboviruses concomitantly could have significant implications for the epidemiology and evolution of these agents. However, our understanding of how *Aedes aegypti* can simultaneously transmit these arboviruses and cause co-infections remains limited^5^. Arboviruses typically cause indistinguishable febrile illnesses, featuring symptoms such as headaches, nausea, arthralgia, and rashes. Therefore, laboratory testing is crucial for accurate diagnosis. Serologies are the standard method for diagnosing arboviruses; however, results may be complicated by antibody cross-reactivity. Molecular techniques, such as reverse transcriptase reaction followed by polymerase chain reaction (RT-PCR) and quantitative real-time PCR (qPCR), are effective and sensitive methods for identifying specific viral genetic material^6^. Accurate and early diagnosis of arboviruses is essential for proper patient management and for the epidemiology of these diseases, enabling the development of potential vaccines, vector control and management strategies, and public awareness campaigns.

Thus, considering the importance of a differential arbovirus diagnosis, this study utilized molecular techniques to detect arbovirus infections and the frequency of co-infections among patients treated at a healthcare center in Goiânia-Goiás, Brazil. Goiânia, the capital of Goiás state, is a city with over 1.5 million residents located in Brazil's Midwest region. 

Initially, patients seeking care at CAIS Jardim Novo Mundo, a public health facility in the eastern part of Goiânia, were screened by the local healthcare team. Those presenting symptoms indicative of an arbovirus infection were selected for the study. All participants were informed about the study's details and provided their consent by signing an Informed Consent Form (ICF). Convenience sampling was employed for participant selection. Blood samples were collected from individuals ≥18 years of age or older and exhibiting symptoms such as an axillary temperature ≥ 37.5°C or a rash, along with two or more concurrent signs or symptoms, including headache, arthralgia, and myalgia. We conducted a cross-sectional study in which 459 blood samples were tested for the arboviruses DENV, ZIKV, CHIKV, and MAYV. These whole blood specimens were collected in clot activator tubes with a separator gel between March 2017 and June 2018. 

After collection, the whole blood samples were transported to the Cytogenetics and Molecular Genetics Laboratory (LGMC) at the Universidade Federal de Goiás in thermal boxes containing dry ice to ensure proper processing. The serum was obtained by centrifuging the samples at 1600 g for 10 minutes. Following centrifugation, aliquots of the sera were stored in nuclease-free cryotubes at -80ºC in an ultra-freezer. All samples underwent RNA extraction using the BioGene® Viral DNA/RNA Extraction Kit K204 (Bioclin, Minas Gerais, Brazil), following the manufacturer’s instructions.

q-PCR for ZIKV, DENV, and CHIKV was performed using the commercial kits BioGene® Zika PCR K203, BioGene® Dengue PCR K201, and BioGene® Chikungunya PCR K202 (Bioclin, Minas Gerais, Brazil), respectively. Reactions were carried out according to the manufacturer's guidelines. An internal control, a plasmid provided in the kits, was added to the samples during RNA extraction. This served as both an RNA extraction and qPCR amplification control. Samples with a threshold cycle (Ct) ≤35 were considered positive.

Conventional PCR was used to detect MAYV in the analyzed samples. Initially, cDNA synthesis was carried out using the M-ML Reverse Transcriptase Kit (Sigma-Aldrich, St. Louis, MO, USA). PCR was then performed to amplify the Nsp1-3 region of MAYV. For primer design, 34 complete MAYV genome strains available in GenBank (NCBI) were aligned using BioEdit (version 7.0.5.2). Primers targeting non-repetitive, evolutionarily conserved genomic regions were designed using the Primer3/BLAST platform at NCBI. The forward and reverse primer sequences were GACGACCTGCAGTCAGTGAT and GTCTTAAAGGCCCACAGGCA, respectively, producing an amplicon of 925 bp. Conventional PCR was performed using the Invitrogen Platinum Taq DNA polymerase (Life Technologies ™, California, USA). Here, 1 µL of cDNA was added to a 25 µL final reaction containing the following: PCR buffer (20 mM Tris HCl (pH 8.4), 50 mM KCl); 2 mM MgCl_2_; 0.2 mM dNTPs; 0.5 μM of each primer; and 2.5 U Platinum Taq DNA Polymerase. For thermocycling, initial denaturation was performed at 95ºC for 5 minutes; subsequently 40 cycles of: denaturation at 95°C for 30 seconds; annealing at 60ºC for 30 seconds and extension at 72ºC for 40 seconds; and then a final extension was performed at 72ºC for 10 minutes. The PCR products were visualized on 1.5% agarose gels.

The results of the 459 samples screened for ZIKV, DENV, CHIKV, and MAYV are summarized in Table 1. DENV was the virus with the largest number of positive cases (130), corresponding to 28.3% of the infected patients; 78 individuals (17%) were positive for MAYV, 58 (12.6%) for ZIKV, and 10 (2.2%) for CHIKV. For technical reasons, it was not possible to perform Q-PCR for ZIKV on 53 samples.

**TABLE 1:** Summary of the results obtained for the molecular tests in patients screened for arboviruses.

	Positive		Negative		Invalid		Not analyzed		Total
	n	%	n	%	n	%	n	%	
DENV	130	28.3	226	49.3	103	22.4	0	0	459
ZIKV	58	12.6	253	55.2	95	20.7	53	11.5	459
CHIKV	10	2.2	436	95.0	13	2.8	0	0	459
MAYV	78	17.0	381	83.0	0	0	0	0	459

DENV: Dengue virus, ZIKV: Zika virus, CHIKV: Chikungunya virus, MAYV: Mayaro virus.

As shown in Figure 1, 49 patients tested positive for more than one arbovirus. The most common co-infection, occurring in 31 (6.7%) of the analyzed samples, was between DENV and ZIKV. Co-infections involving DENV and MAYV were observed in 10 (2.2%) samples. Less frequent co-infections included CHIKV and MAYV (3 patients, 0.7%) and ZIKV and MAYV (2 patients, 0.4%). Additionally, three patients were simultaneously co-infected with three arboviruses: ZIKV, DENV, and MAYV.

**FIGURE 1:**
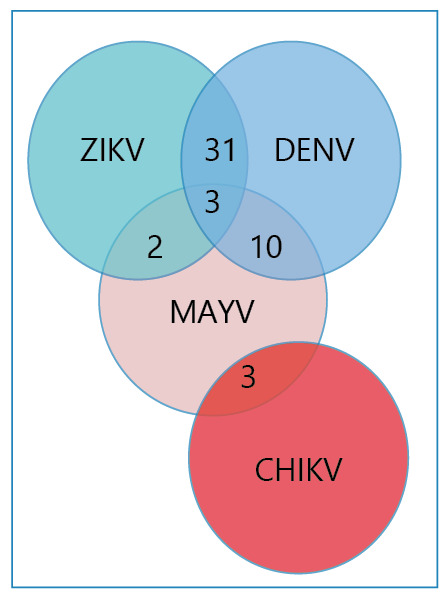
Venn diagram representing arbovirus co-infections in screened patients. **DENV:** Dengue virus; **ZIKV:** Zika virus; **CHIKV:** Chikungunya virus; **MAYV:** Mayaro virus.

Brazil periodically experiences arboviral outbreaks, particularly during the rainy season. The incidence of these diseases varies by region and year, with some areas being more prone to outbreaks. Goiás has reported a significant number of cases annually. According to the Goiás State Health Department, 173,410 cases of arboviral infections were reported from 2017 to 2023. Of these, 10,110 (5.83%) were due to ZIKV, 13,856 (7.99%) to CHIKV, and 149,444 (86.18%) to DENV. Several deaths related to these infections have also been reported, especially due to DENV^7^. Our results indicate that DENV infections were the most prevalent, followed by CHIKV and ZIKV infections. However, we did not observe a significant discrepancy in the percentage of dengue cases compared to the data released by state health authorities. A higher number of dengue notifications may reflect an under-testing for other arboviruses. Unfortunately, neither public nor private healthcare systems are adequately equipped to diagnose these arboviruses rapidly and efficiently, nor to differentiate between them.

Infections with more than one arbovirus have been described in Brazil^8^ ^,^ ^9^ and Colombia^10^. Arbovirus co-infection is commonly reported in endemic areas where transmission rates are significant^8^ ^-^ ^11^. Thus, the Pan-American Health Organization recommends screening samples using consecutive assays as soon as a pathogen is identified^3^. However, the Centers for Disease Control and Prevention (CDC) recommends simultaneous qPCR for the detection of DENV, CHIKV, and ZIKV^12^.

Although arboviruses commonly cause benign diseases, their symptoms can persist for several weeks. Moreover, severe cases can lead to irreversible sequelae such as microcephaly caused by ZIKV. It is important to note that the co-circulation of arboviruses presents challenges for clinical and laboratory diagnoses in endemic areas. Patients infected with one or more viruses may exhibit similar clinical manifestations of viremia. Additionally, due to frequent co-infections, it is crucial to test patients for each virus to ensure accurate and sensitive diagnosis, which is essential for clinical management, research, and epidemiological surveillance of arboviruses^13^. Furthermore, the co-circulation of MAYV with DENV has been observed in the population of Goiânia^14^, underscoring the need for effective laboratory diagnostic assays to identify infections. Therefore, confirming arbovirus infection using sensitive laboratory diagnostic methods is essential to avoid misrepresentative results, which may lead to inadequate management of infections and contribute to the epidemiological surveillance of arboviruses.

Molecular diagnostics play a significant role in the detection, quantification, and typing of viruses. The main advantages of PCR and qPCR are their speed, reliability, and capacity for high-throughput detection of target sequences. These techniques exhibit good sensitivity and specificity. However, each method has inherent limitations. The principal challenges of applying PCR and qPCR assays in clinical settings include false-positive results due to background DNA contamination and the potential for false-negative results. In this study, we employed good laboratory practices to minimize these limitations. For instance, reactions and samples were pipetted in separate rooms using a laminar flow hood. An internal control was added to the samples during RNA extraction to serve as both an RNA extraction and amplification control.

Thus, the significance of this study lies in its contributions to arbovirus surveillance and control. Fast and accurate diagnostic tools are crucial for ensuring patients receive appropriate treatment and are vital for informing public health system policies, as well as for aiding in epidemiological surveillance.
